# Endocytosis at extremes: Formation and internalization of giant clathrin-coated pits under elevated membrane tension

**DOI:** 10.3389/fmolb.2022.959737

**Published:** 2022-09-21

**Authors:** Ahmet Ata Akatay, Tianyao Wu, Umidahan Djakbarova, Cristopher Thompson, Emanuele Cocucci, Roya Zandi, Joseph Rudnick, Comert Kural

**Affiliations:** ^1^ Department of Physics, The Ohio State University, Columbus, OH, United States; ^2^ Interdisciplinary Biophysics Graduate Program, The Ohio State University, Columbus, OH, United States; ^3^ Division of Pharmaceutics and Pharmacology, College of Pharmacy and Comprehensive Cancer Center, The Ohio State University, Columbus, OH, United States; ^4^ Department of Physics and Astronomy, University of California, Riverside, CA, United States; ^5^ Department of Physics and Astronomy, University of California, Los Angeles, CA, United States

**Keywords:** clathrin, endocytosis, superresolution microscopy, embryogenesis, mechanobiolgy, adhesion

## Abstract

Internalization of clathrin-coated vesicles from the plasma membrane constitutes the major endocytic route for receptors and their ligands. Dynamic and structural properties of endocytic clathrin coats are regulated by the mechanical properties of the plasma membrane. Here, we used conventional fluorescence imaging and multiple modes of structured illumination microscopy (SIM) to image formation of endocytic clathrin coats within live cells and tissues of developing fruit fly embryos. High resolution in both spatial and temporal domains allowed us to detect and characterize distinct classes of clathrin-coated structures. Aside from the clathrin pits and plaques detected in distinct embryonic tissues, we report, for the first time, formation of giant coated pits (GCPs) that can be up to two orders of magnitude larger than the canonical pits. In cultured cells, we show that GCP formation is induced by increased membrane tension. GCPs take longer to grow but their mechanism of curvature generation is the same as the canonical pits. We also demonstrate that GCPs split into smaller fragments during internalization. Considering the supporting roles played by actin filament dynamics under mechanically stringent conditions that slow down completion of clathrin coats, we suggest that local changes in the coat curvature driven by actin machinery can drive splitting and internalization of GCPs.

## 1 Introduction

Clathrin-coated vesicles are the fundamental functioning units of lipid and protein trafficking from the plasma membrane to endosomes (Conner and Schmid, 2003). They are formed by gradual recruitment of heterohexameric clathrin triskelions to the plasma membrane by a diverse set of adaptor proteins that bind to clathrin, membrane, and other adaptor and accessory proteins (Taylor et al., 2011; Kural and Kirchhausen, 2012). Clathrin triskelions assemble into polyhedral cages (100–200 nm in diameter) that generate endocytic pockets on the plasma membrane (Heuser, 1980), altogether referred as clathrin-coated pits (CPs). As the membrane neck linking the CP to the plasma membrane gets narrower, dynamin is recruited to this region in a burst and drives membrane scission that leads to internalization of a clathrin-coated vesicle (Cocucci et al., 2014).

Membrane tension is a fast, effective and reversible regulator of endocytic clathrin-coated vesicle formation (Ferguson et al., 2016, Ferguson et al., 2017; Willy et al., 2017; Djakbarova et al., 2021; Willy et al., 2021a). Increased membrane tension reduces the initiation density (Ferguson et al., 2017), and slows down the growth and dissolution rates of endocytic clathrin-coated structures (Ferguson et al., 2016). Moreover, internalization of CPs from the plasma membrane becomes dependent on the curvature generating adaptor proteins (Joseph et al., 2020; Willy et al., 2021a) and the forces provided by actin polymerization under increased tension (Boulant et al., 2011; Jin et al., 2022; Kaplan et al., 2022).

Clathrin-coated vesicles can originate through different mechanisms. The best characterized mechanism is based on formation of CPs *de novo*, where the initiation, maturation, and internalization of the CP is independent and spatially isolated from other clathrin-coated structures (Ehrlich et al., 2004; Cocucci et al., 2012; Aguet et al., 2013; Willy et al., 2021b). In the second mechanism, large clathrin lattices found at membrane-substrate adhesion sites, i.e., clathrin plaques, serve as CP initiation sites (Lampe et al., 2016; Leyton-Puig et al., 2017). It is proposed that highly curved CPs forming at the edges of relatively flat plaques increase the strain on the clathrin lattice, eventually giving rise to ruptures and breaks that allow growth and internalization of CPs independently (den Otter and Briels, 2011; Willy et al., 2021b).

Here, we studied the dynamics of endocytic clathrin-coated structures within live cultured cells and *Drosophila* embryos using super-resolved fluorescence imaging. During our comprehensive studies of clathrin coated vesicle formation, we identified new structures that we named giant coated vesicles (GCPs) first in cultured cells then in tissues of developing *Drosophila* embryos. In cultured cells, GCPs were only observed when the membrane tension was increased by hypotonic swelling or cholesterol depletion (Biswas et al., 2019). Interestingly, GCPs were internalized by splitting into multiple fragments comparable in size to the canonical CPs, suggesting a new mechanism of endocytic clathrin-coated vesicle formation. In the epidermal tissue of late-stage *Drosophila* embryos, we found that GCPs can be up to two orders of magnitude larger than the canonical CPs observed in the same tissue. Similar to the “rupture and growth” model suggested for CP formation at the edges of clathrin plaques, we propose a model where GCPs go through splitting events initiated by the forces provided by the actin machinery giving rise to increased strain and breaks on the clathrin coat.

## 2 Results

### 2.1 Formation and internalization of canonical clathrin-coated pits (CPs)

**FIGURE 1:**
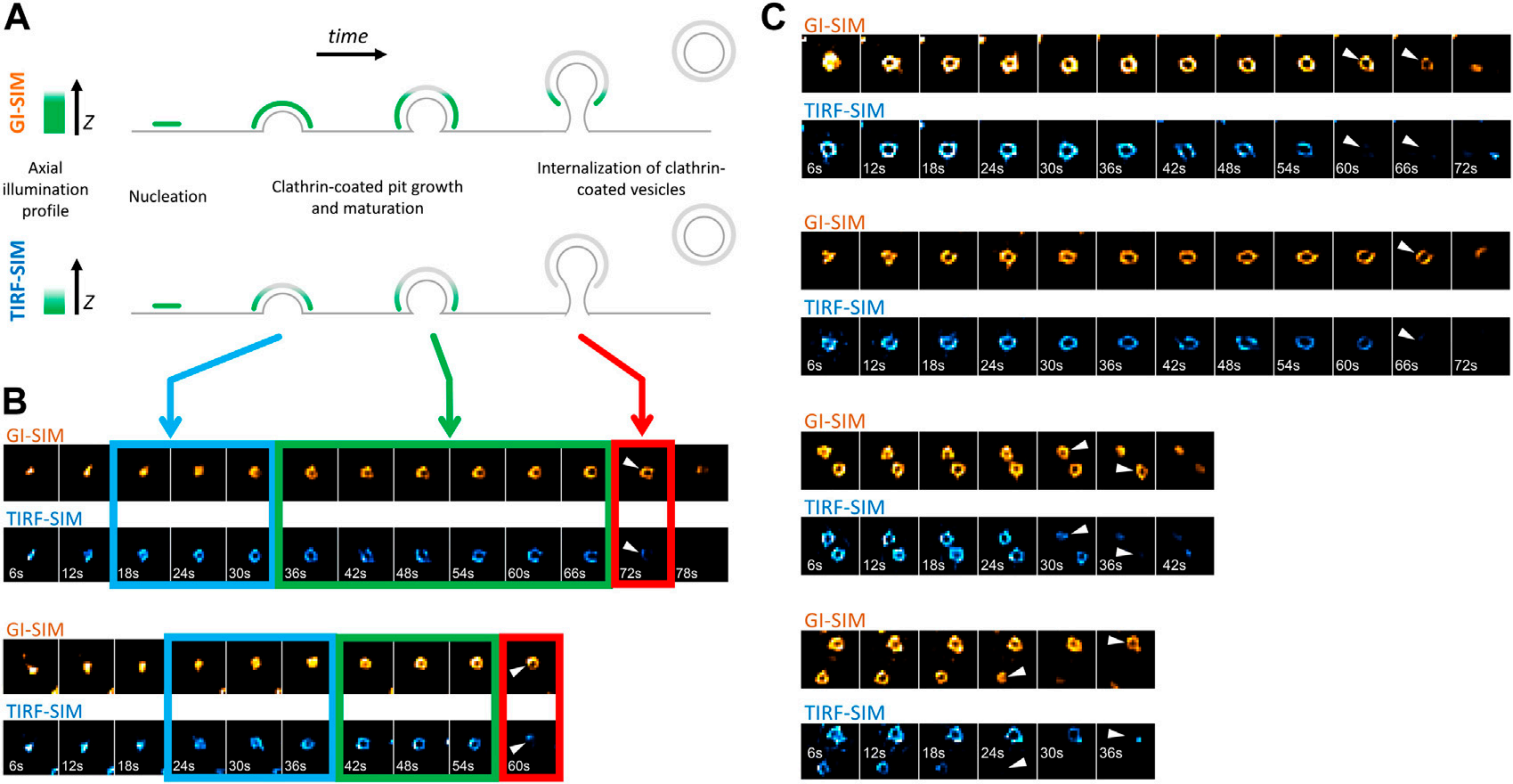
Formation and internalization of “canonical” clathrin-coated pits (CPs) as detected by simultaneous high NA TIRF-SIM & GI-SIM imaging in living cells. **(A)** GI-SIM offers longer penetration depth, i.e., fluorescence excitation along the *z*-dimension, compared to TIRF-SIM. As invagination of the endocytic pit increases, the apex of the clathrin coat exits the excitation field of TIRF-SIM earlier, resulting in emergence of the ring pattern (blue boxes in B). As the coat matures into a pit, it is observable as a ring in both channels (green boxes in B). While the coat is internalized as a whole, it leaves the TIRF field but still can be observed in the GI-SIM channel (red boxes in B). **(B)** Montages show formation and internalization of two CPs as imaged by GI-SIM and TIRF-SIM simultaneously in SUM159 cells genome edited to express AP2-EGFP. **(C)** More examples displaying the inward movement of CPs and their internalization as a whole, i.e., without splitting into multiple fragments. Arrowheads mark disappearance of the clathrin pits from the TIRF-SIM channel while they are still observable by GI-SIM. Each region of interest in B and C, 1 µm × 1 µm.

Formation, internalization and dissolution dynamics of endocytic clathrin coats have been characterized in living cells by studies utilizing fluorescence microscopy (Cocucci et al., 2012; Kural and Kirchhausen, 2012; Cocucci et al., 2014; Aguet et al., 2016; Willy et al., 2021a). In these assays, CPs appear as featureless diffraction-limited spots as their typical size is smaller than the resolution limit of the conventional fluorescence microscope. Nevertheless, super-resolved fluorescence microscopy allows to elucidate the structural properties of endocytic CPs, along with their dynamics (Jones et al., 2011; Fiolka et al., 2012; Li et al., 2015). In particular, structured illumination microscopy at the total internal reflection fluorescence mode (TIRF-SIM) obtained with high numerical aperture (NA) objectives has been instrumental in monitoring curvature generation by endocytic clathrin pits within live cells and tissues (Willy et al., 2021b). In these acquisitions, the total internal reflection of the excitation beam creates an evanescent field that illuminates the lateral regions of CPs at a higher intensity compared to the apex, which is further away from the glass substrate. As a result, formation of CPs is marked by a characteristic “ring” pattern (100–200 nm in diameter) under super-resolution imaging (Li et al., 2015; Joseph et al., 2020; Willy et al., 2021b) (Figures 1A,B). Reducing the excitation NA (i.e., incidence angle of the excitation beam) enables increasing the penetration depth of the illumination field, i.e., approaching the structured illumination microscopy at the grazing incidence mode (GI-SIM), and imaging deeper inside cultured cells and fruit fly embryos with enhanced spatiotemporal resolution (Guo et al., 2018). Here, we monitored formation of *de novo* CPs in SUM-159 cells genome edited to express AP2-EGFP (Aguet et al., 2016) using high NA TIRF-SIM and GI-SIM imaging simultaneously to follow the growth of the coat in the axial dimension (i.e., along *z*) while monitoring curvature generation in real time. We found that the ring pattern arose in the TIRF-SIM channel soon after the nucleation of the coat (Figure 1B- blue boxes). However, due to the longer penetration depth of GI-SIM, the same pattern was not visible in this channel in this time window. As the coat grew further and matured into a pit, the ring pattern was observed in both TIRF-SIM and GI-SIM channels (Figure 1B- green boxes). Altogether, these results demonstrate that the coat is highly curved even at the early stages of clathrin pit formation as proposed earlier and, since the ring pattern appears following an increase in the footprint of the coat, curvature generation does not depend on a flat-to-curved transition (Chen et al., 2019; Willy et al., 2021b). Interestingly, at later stages, the ring pattern disappeared in the TIRF-SIM channel but persisted in the GI-SIM channel, demonstrating the inward movement (along the axial dimension) and internalization of the clathrin pit as a whole (Figure 1B- red boxes, Figure 1C). It was previously demonstrated that the inward movement of endocytic CPs during their internalization is dependent on actin polymerization (Ferguson et al., 2017). Moreover, it has been shown that actin-driven displacement of clathrin coats (away from the cell surface) gets longer with increasing membrane tension (Ferguson et al., 2017), in accord with models suggesting that actin polymerization pushing the clathrin coats increases with the increasing mechanical load (Kaplan et al., 2022).

### 2.2 New mechanisms of clathrin coat internalization emerge under high mechanical load

**FIGURE 2:**
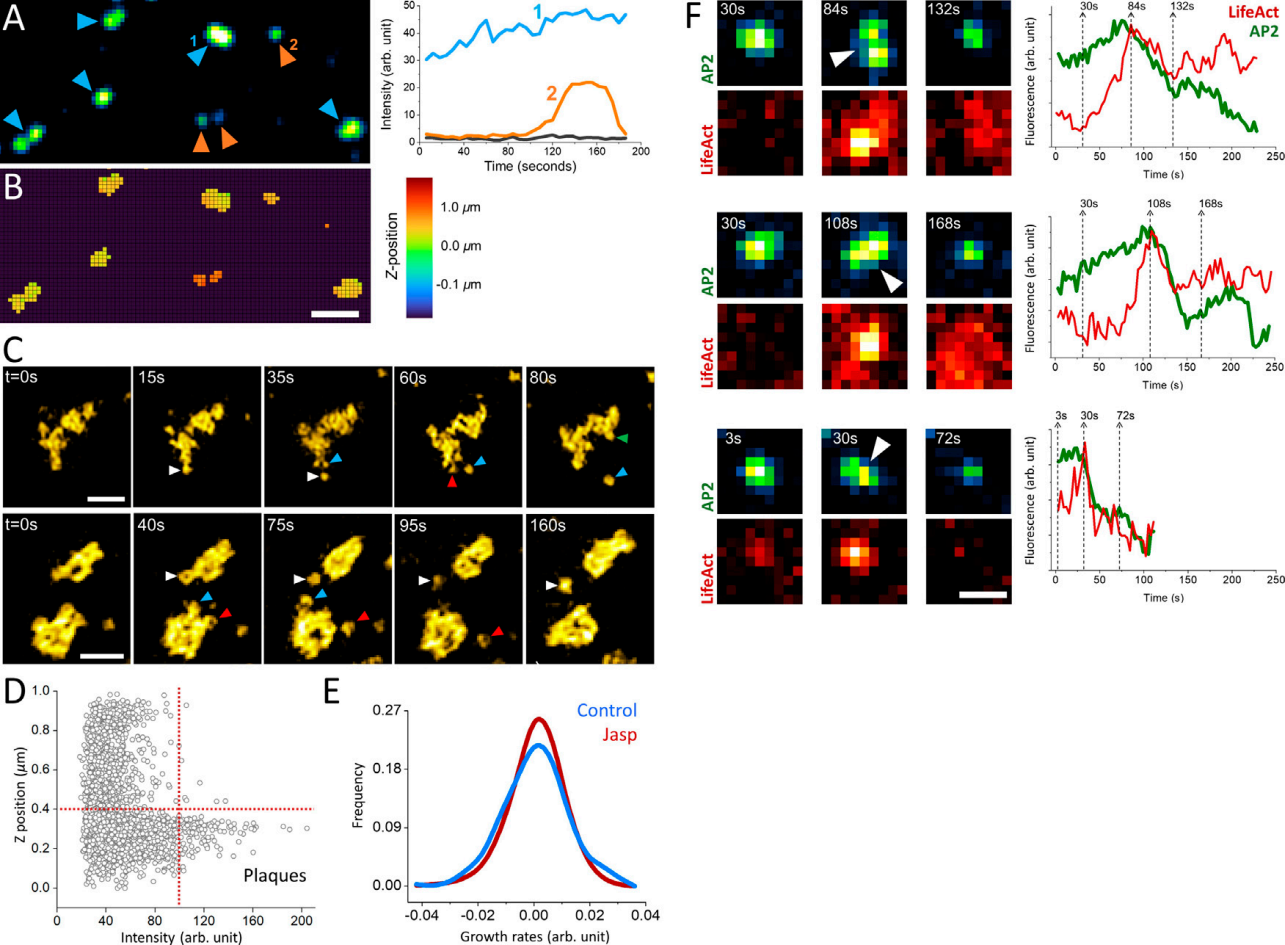
Role of actin in clathrin coat dynamics under high mechanical load. **(A)** Clathrin-coated pits and plaques are imaged on the ventral surface of live SUM-159 cells using spinning-disk confocal microscopy. The blue and orange arrowheads mark plaques and pits, respectively. Representative fluorescence intensities of a clathrin plaque (1) and pit (2) are shown on the left panel, where the black line shows the background intensity. Although its intensity has changed over time, the clathrin plaque was observable throughout the entire acquisition. Whereas the clathrin pit in the example had a lifetime of ∼100 s, it appeared around the 90th second and was internalized at the end of the acquisition. **(B)** The average z-positions of the pits and plaques in **(A)** are determined for each pixel as described earlier (Kural et al., 2012). Plaques are localized to membrane-substrate adhesion sites and, therefore, are found in the same axial position. Whereas the clathrin pits are often observed at higher z positions (Ferguson et al., 2016). Scale bar, 1 µm. **(C)** Examples show endocytic pits (marked by arrowheads with distinct colors) budding off from large clathrin plaques. Scale bars, 1 µm. **(D)** Scatter plot shows the relative z-position versus the maximum intensity of clathrin-coated structures detected at the ventral surface of SUM-159 cells genome edited to express AP2-EGFP (N_cells_ = 2; N_traces_ = 3736). The dashed lines denote the thresholds used to select plaques, which are brighter than pits and positioned closer to the substrate (Ferguson et al., 2016). **(E)** After plaques are identified as described in **(D)**, their growth rate distributions are plotted prior to (Control, N_TraceFragments_ = 365) and 10 min after treatment with 1 µM Jasplakinolide (Jasp, N_TraceFragments_ = 1944). The narrower growth distribution upon Jasp treatment is a result of slower plaque dynamics upon inhibition of actin machinery (Ferguson et al., 2016). **(F)** Increased membrane tension upon cell squeezing gives rise to formation of large and bright clathrin puncta, which can split in multiple fragments that are internalized independently. Three such events are shown in both AP2 (to mark clathrin coats) and Lifeact (actin marker) channels, where the corresponding integrated fluorescence intensities are shown on the right. The dashed lines show the corresponding time points. The arrowheads mark the splitting events that coincide with a burst in actin recruitment that is followed by a significant reduction in the AP2 signal. Scale bars, 0.5 µm.

Actin dynamics provide the energy required for internalization of clathrin-coated vesicles under mechanically stringent conditions that slow down completion of clathrin coats (Boulant et al., 2011; Ferguson et al., 2017; Kaplan et al., 2022). For instance, uptake of viral particles larger than the canonical clathrin pits take longer than the time required for internalization of smaller cargo molecules, and necessitate a “push” provided by actin polymerization that moves the clathrin coat away from the plasma membrane (Cureton et al., 2010). In certain cell types, the adhesion between the plasma membrane and the substrate slow down the endocytic machinery and result in the formation of clathrin plaques, large and flat clathrin lattices that are longer lived than the canonical CPs (Batchelder and Yarar, 2010; Ferguson et al., 2016; Lampe et al., 2016). In three-dimensional fluorescence live cell imaging assays, clathrin plaques appear as bright and long-lived puncta localized at cell-substrate adhesion sites (Figures 2A,B). The edge regions of clathrin plaques are known as actin-dependent endocytic hubs (Lampe et al., 2016; Leyton-Puig et al., 2017), where local mismatches in coat curvature are proposed to give rise to ruptures in the clathrin lattice and allow individual CPs to split from the plaque and internalize independently (den Otter and Briels, 2011; Willy et al., 2021b) (Figure 2C). It was proposed that polymerization of actin filaments generates the “pushing” force required for internalization of plaques from the plasma membrane (Saffarian et al., 2009). Indeed, we found that the growth and dissociation dynamics of clathrin plaques, represented by positive and negative growth rates (Ferguson et al., 2016, Ferguson et al., 2017; Willy et al., 2017), reduce in SUM-159 cells when actin machinery is inhibited by a mild Jasplakinolide treatment (Boulant et al., 2011; Kural et al., 2015) (Figures 2D,E).

Increased membrane tension is another factor that renders internalization of clathrin coats dependent on the forces generated by actin polymerization (Boulant et al., 2011; Kaur et al., 2014; Kaplan et al., 2022). When the plasma membrane tension is increased by squeezing cells using a soft polymer cushion, dynamic CPs are replaced by bright fluorescent puncta corresponding to large and long-lived clathrin-coated structures as imaged under diffraction-limited fluorescence microscopy (Ferguson et al., 2017). We found that splitting events can be observed in this population of clathrin-coated structures and characterized by increased eccentricity of the fluorescent spot followed by internalization of a portion of the coat and a significant reduction in integrated fluorescence signal (Figure 2F). Splitting events coincided with a burst of actin fluorescence, suggesting that actin polymerization provides the force necessary for splitting of large clathrin coats into smaller pieces prior to internalization. We decided to investigate these events further using super-resolution microscopy to elucidate clathrin coat dynamics under increased membrane tension.

### 2.3 Increased membrane tension induces formation of giant coated pits (GCPs) in cultured cells

**FIGURE 3:**
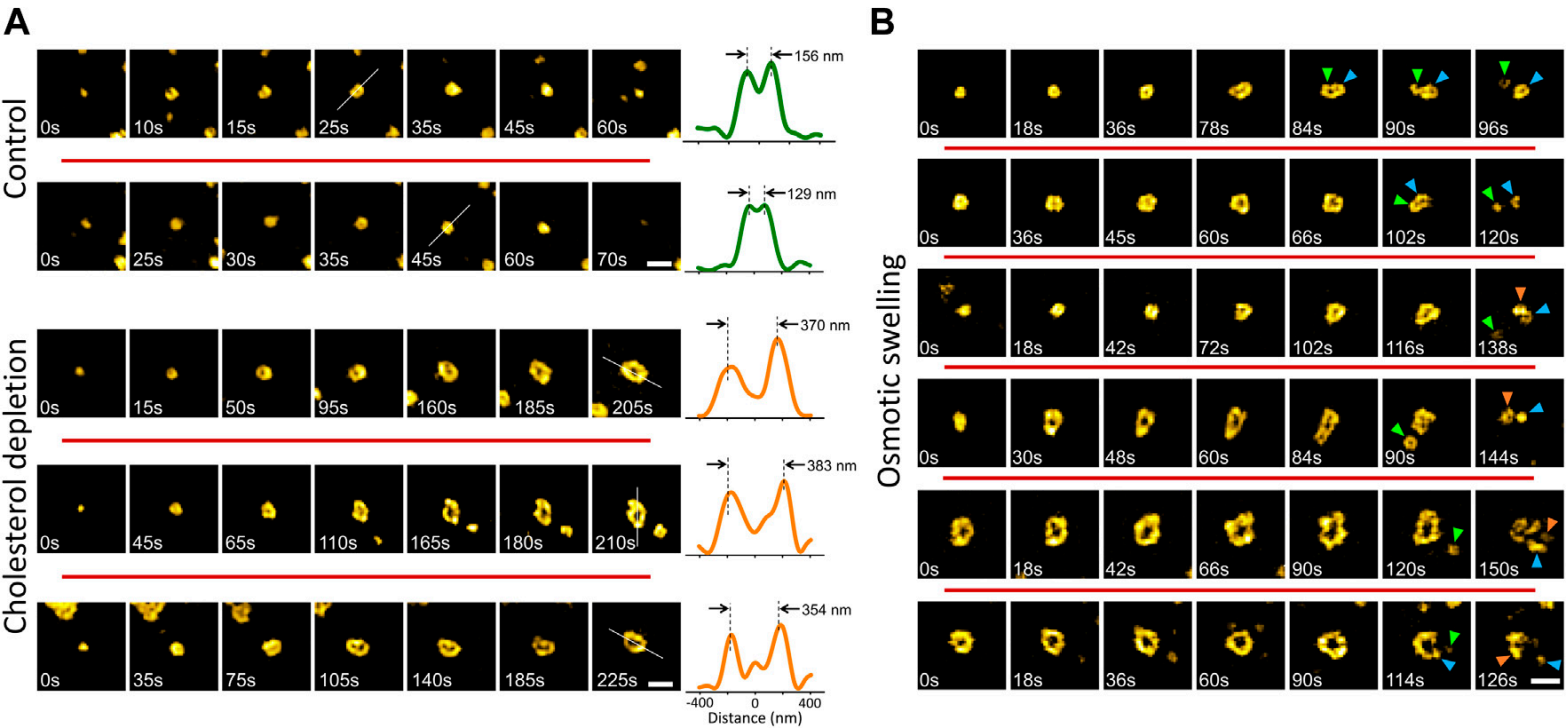
Formation and dissolution of giant coated pits (GCPs) in cultured cells. **(A)** Examples (in different rows) of canonical CP formation detected in live SUM159 cells expressing clathrin-mRuby (upper). Cholesterol depletion by methyl-β-cyclodextrin (MβCD) treatment induces GCP formation in the same cell line (lower). For each structure, the intensity profile along the dashed line is plotted on the right. Peak-to-peak distances demonstrate the size difference between CPs and GCPs. **(B)** Examples (in different rows) of GCP formation and dissolution detected upon hypotonic swelling in SUM159 cells genome edited to express AP2-EGFP. Arrowheads in different colors mark segments separated from the GCPs. Scale bars, 0.5 µm.

Membrane tension affects the dynamics and structure of endocytic clathrin coats by increasing the energy cost of curvature generation on the plasma membrane (Heuser, 1989; Subtil et al., 1999; Boulant et al., 2011; Ferguson et al., 2016, 2017; Willy et al., 2017; Willy et al., 2021a; Willy et al., 2021b). Here, we employed two independent strategies, compatible with TIRF-SIM imaging, to increase the mechanical load on the endocytic clathrin machinery. First, we treated cultured cells with methyl-β-cyclodextrin (MβCD) to deplete cholesterol from the plasma membrane (Ferguson et al., 2016; Willy et al., 2017; Biswas et al., 2019). Second, we used hypotonic medium to induce osmotic swelling in cells (Dai et al., 1998; Ferguson et al., 2017). We found that increased membrane tension due to cholesterol depletion gives rise to formation of giant coated pits (GCPs) that are substantially larger than the canonical CPs (∼8 times greater in volume given that the radius is more than 2 times larger; Figure 3A). Even though GCP formation is markedly longer than the canonical CPs, the mechanism of curvature generation is the same: the footprint of the coat increased with the detection of the ring pattern, which indicates that the GCP curvature is generated at early stages of the coat formation without a flat-to-curved transition that requires restructuring of the coat (Willy et al., 2021b) (Figure 3A).

To induce osmotic swelling we exposed cells to 80% hypotonic medium (Willy et al., 2021a). In contrast to cholesterol depletion, osmotic swelling and its effects on the plasma membrane tension and clathrin-mediated endocytosis dynamics is temporary (Dai et al., 1997; Ferguson et al., 2017). We observed that GCPs are formed and internalized within the first 15 min of the hypo-osmotic treatment, where the membrane tension is expected to be the highest (Dai et al., 1997; Bucher et al., 2018; Willy et al., 2021b). Interestingly, we found that GCPs observed in swollen cells split into multiple small pieces, some of which have the characteristic ring pattern of the canonical CPs (Figure 3B) (Supplementary Video S1).

### 2.4 Giant coated pits (GCPs) are present in the epidermis of late-stage *Drosophila* embryos

**FIGURE 4:**
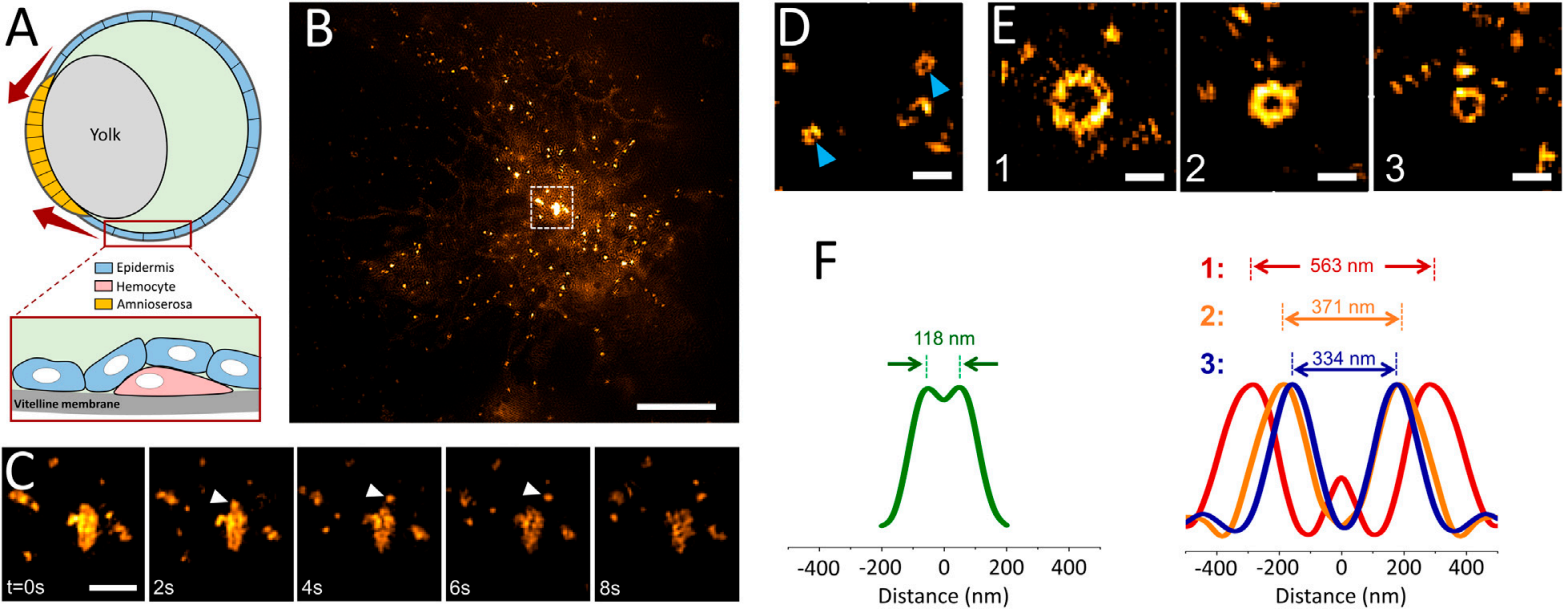
Multifarious assemblies of clathrin imaged in tissues of *Drosophila* embryos. **(A)** Schematics depicting the cross-section of a late-stage *Drosophila* embryo during dorsal closure. *Drosophila* hemocytes (pink) can be imaged by TIRF-SIM as they migrate through the space between the vitelline membrane and epidermis (blue) as seen in Supplementary Video S3. The red arrows represent the increasing tension at the apical surface of the amnioserosa-epidermis interface throughout the dorsal closure. **(B)** TIRF-SIM image of a Drosophila hemocyte expressing clathrin-mEmerald right above the vitelline membrane of a live embryo. Scale bar, 5 µm. **(C)** Montage shows the boxed region in **(B)**, where the arrowheads mark a coat fragment splitting from a clathrin plaque. Scale bar, 1 µm. **(D)** Arrowheads mark two canonical CPs imaged within developing *Drosophila* embryos expressing clathrin-mEmerald. Scale bar, 0.5 µm. **(E)** GCPs imaged at the epidermis tissue during dorsal closure of the embryo. Scale bars, 0.5 µm. **(F)** Left: Radial average of canonical clathrin pit images obtained from embryos, where the peak-to-peak distance is 118 nm. Right: Radial averages of the three GCPs shown in **(E)**, where the corresponding peak-to-peak separations are 563, 371 and 334 nm, respectively.

Since GCPs were observed in cultured cells only under increased membrane tension, we hypothesized that such structures can form in distinct tissues of multicellular organisms, particularly during developmental stages associated with generation of mechanical forces and increased tension (Kiehart et al., 2017). Reducing the excitation NA in TIRF-SIM acquisitions enables monitoring processes deep inside the specimen (Figure 1), including formation of individual clathrin-coated structures within tissues of developing *Drosophila* embryos expressing fluorescently tagged clathrin coat components (Willy et al., 2021b) (Supplementary Video S2). In these *in vivo* acquisitions, we have detected formation of clathrin plaques on migratory hemocytes that occasionally move adjacent to the vitelline membrane of the embryo (Figures 4A,B; Supplementary Video S3). Similar to our observations in cultured cells (Figure 2C), we observed clathrin plaques detected *in vivo* also go through splitting events that give rise to smaller coat fragments internalized independently from the edge regions (Figure 4C).

Mechanical forces play central roles during the dorsal closure of the *Drosophila* embryo, resulting in increased tension in the amnioserosa and lateral epidermis tissues (Figure 4A) (Saias et al., 2015; Hayes and Solon, 2017). In good agreement with our hypothesis, we detected formation of clathrin domes that are significantly larger than the canonical CPs on the apical surface of epidermal cells of late-stage *Drosophila* embryos (Figures 4D,E; Supplementary Video S3). We found that the diameter of these GCPs reaches up to 0.5 µm, about 5-times wider than the typical size of CPs (i.e., more than 100-times larger in volume) observed within the same tissue (Figure 4F). Similar to our findings in cultured cells, a subset of GCPs were split into multiple fragments before internalization (Supplementary Video S4). As the next step, we decided to scrutinize the factors that can result in splitting of GCPs.

### 2.5 Simulating the dissolution of GCPs

In cell culture, formation of GCPs is induced by increased membrane tension. Unlike osmotic swelling experiments, GCPs forming upon MβCD treatment were stalled and did not undergo splitting and dissolution. Note that cholesterol depletion and osmotic swelling increase the effective membrane tension in different ways. Cholesterol depletion not only affects the rigidity of the plasma membrane but also increases the adhesion of actin cytoskeleton to the plasma membrane (Khatibzadeh and Gupta, 2012), which is expected to impact actin filament dynamics. Whereas osmotic swelling increases the in-plane membrane tension without affecting the function of actin machinery (Dai et al., 1998; Boulant et al., 2011; Ferguson et al., 2017; Kaplan et al., 2022). Considering the fact that actin machinery becomes indispensable for internalization of clathrin-coated vesicles under high mechanical load (Boulant et al., 2011; Ferguson et al., 2017), we propose that splitting and internalization of GCPs under high membrane tension is also dependent on the pushing forces provided by actin polymerization. Similar to the ‘rupture and growth” model proposed for plaques, local changes in the GCP curvature imposed by actin polymerization is expected to increase the strain and give rise to breaks on the coat (den Otter and Briels, 2011; Lampe et al., 2016; Willy et al., 2021b).

**FIGURE 5:**
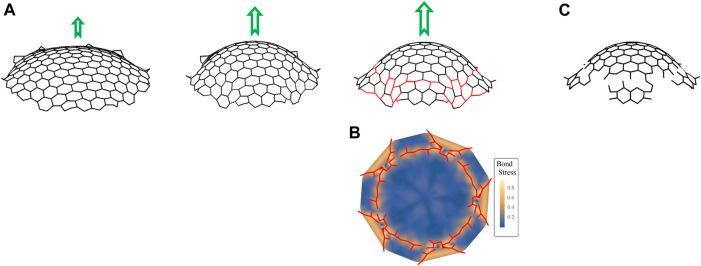
Simulations of GCP segments. **(A)** Snapshots of simulations showing the evolution of a clathrin complex grown from a sheet of hexagons surrounding a central pentagon into a dome with increasing force (represented by green arrows). The parameters used in simulations are $k_{s}=500,$ $l_{0}=0.64,$ $k_{b}=10^{-8}$ , $k_{p}=100$ and $\theta_{0}=2\pi /3$ . Red lines in the final stage of the dome denote the bonds that break due to bond stress exceeding the threshold of 0.3. **(B)** The bond stress map $S_{B}$ of the final stage of the dome, as given by (Eq. A.6), as viewed from above. **(C)** The same clathrin coat with the broken bonds removed, along with small, detached fragments.

To test how increasing curvature can affect the local strain on a GCP segment, we simulated the transformation of a clathrin lattice containing a pentagonal defect to a dome using a steepest descent technique, in which we calculated the total energy of each vertex and moved the vertices as follows: $\frac{{dp}_{i}\left(t\right)}{dt}=-\gamma \frac{\partial E\left(p_{1}\left(t\right),p_{2}\left(t\right),\ldots \right)}{\partial p_{i}\left(t\right)}$ where *p*_*i*_(*t*) is the position of each vertex of triskelion network (see Figure 5A), *γ* the relaxation rate that we set equal to 1. The total energy *E* depends on the state of triskelions: stretched or compressed (Eq. A.2 in Material and Methods section), the angle between adjacent edges of a hexagon (Eq. A.1) and the “pucker angle” or local curvature energy (Eq. A.3)*,* see the materials and methods section for the details. Our system then depends on a few moduli (stretching $k_{s}$ , bending $k_{b}$ , curvature $k_{p}$ ), the equilibrium length of edges ( $l_{0})$ and the preferred curvature $\left(C_{0}\right).$ All the parameters in the model are constant except $C_{0}$ , which can vary with time for multiple reasons. For example, as the tension in the cell decreases, the membrane below the clathrin lattice becomes floppier and thus occupies more volume. A membrane under tension favors a smaller positive value for $C_{0}$ , while as the substrate becomes flaccid $C_{0}$ increases. The variation of $C_{0}$ with time could also be due to pushing forces provided by actin polymerization (Figure 5A). Regardless of the sources, in our model, we consider the influence of these forces through the change in the value of $C_{0}$ , allowing the coat curvature to increase.

We then solve the equation of motion given in the previous paragraph and (Eq. A.5), with parameters in the total energy adjusted so that there is an energetic preference for a constant curvature. This is done by setting the parameter $C_{0}$ equal to a time dependent value that increases linearly with time from 0 at t=0 to 0.04 at the final time, which we set equal to $10^{8}$ . All other parameters in the equation for the total energy are given time-independent values. In particular, we set $k_{s}=500$ and $l_{0}=0.64$ in (Eq. A.2), $k_{b}=10^{-8}$ in (Eq. A.1), $k_{p}=100$ in (Eq. A.3) and $\theta_{0}=2\pi /3$ . The last assignment gives rise to a contribution to the energy that favors regular hexagons. The result of our simulations is shown in Figure 4A, which shows snapshots of how the lattice distorts as the strength of the force that induces curvature on the complex increases. The bond stress map $S_{B}$ (see Eq. A6 in the materials and methods) in Figure 5B reveals that the stress is in particular high at the edge. The bonds break if bond stress exceeds the threshold of 0.3 (shown as red lines in the stress map). Overall, as suggested by the “rupture and growth” model, the deformations resulting at the edges of the coat lead to increased strains in these regions, which may result in breaks in the lattice leading to fragmentation of the GCP (Figure 5C) and, subsequently, growth and internalization of CPs independently.

## 3 Discussion

**FIGURE 6:**
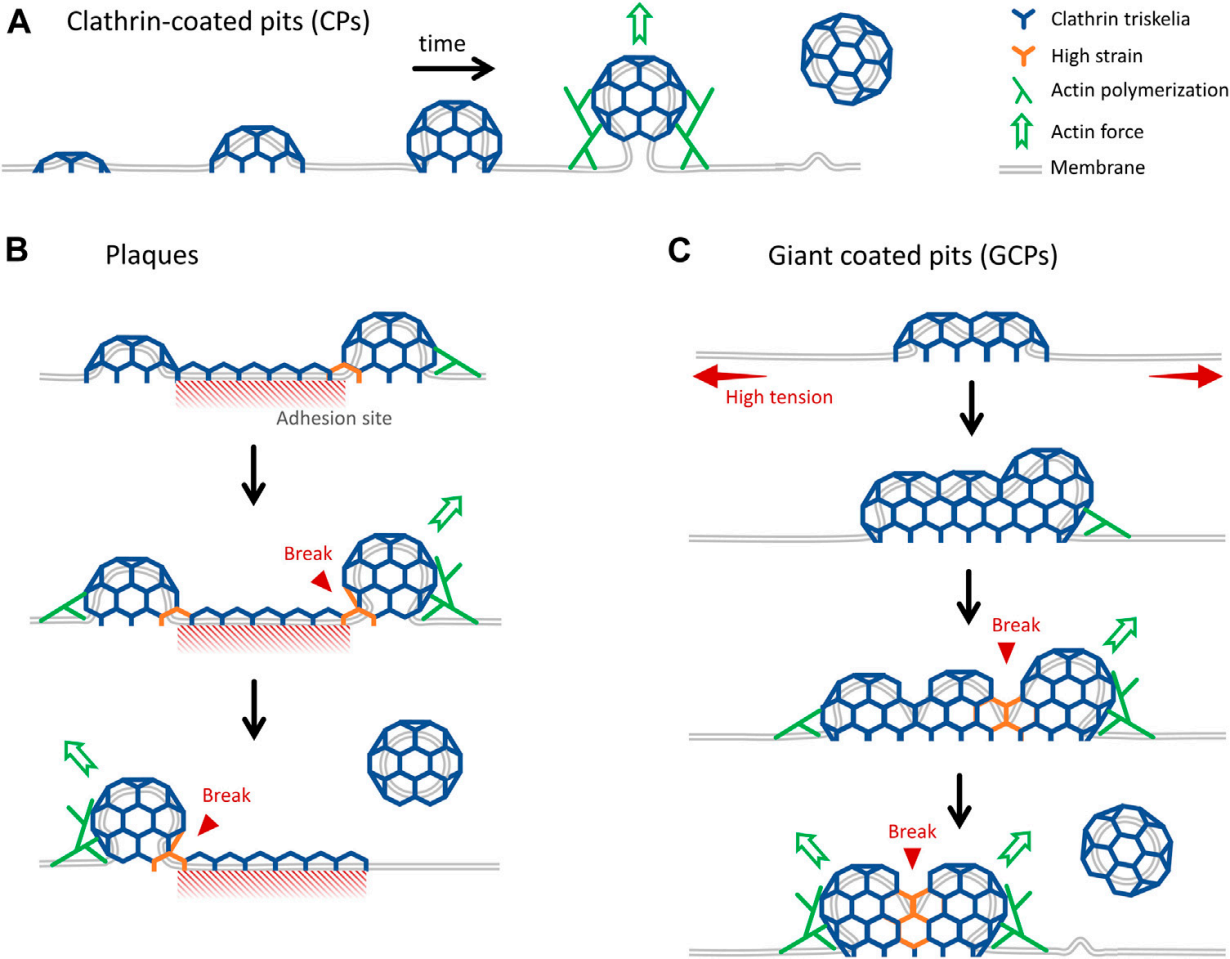
Distinct mechanisms of internalization of endocytic clathrin-coated vesicles. **(A)** Cartoon representation of growth and internalization of canonical CPs. The inward movement of the pit during internalization is dependent on actin dynamics, which becomes indispensable under increased membrane tension (Boulant et al., 2011; Ferguson et al., 2017). **(B)** Clathrin plaques are predominantly observed at the membrane-substrate adhesion sites. It was proposed that clathrin pits can break apart from the edges of plaques and grow into coated vesicles independently. This “rupture and growth” model proposes that increased local curvature creates strain on the lattice, giving rise to breaks and splitting coat fragments (den Otter and Briels, 2011; Lampe, Vassilopoulos and Merrifield, 2016; Willy et al., 2021b). **(C)** GCPs form under increased membrane tension. They are larger than CPs, but they impose membrane curvature in a similar way. We propose that forces produced by actin polymerization, aided by reducing plasma membrane tension, give rise to splitting of GCPs into multiple small fragments that are internalized independently.

In this study, we developed and used advanced super-resolution imaging modalities to study the formation and internalization dynamics of distinct classes of endocytic clathrin-coated structures within cultured cells and tissues of developing *Drosophila* embryos. We have focused on three different mechanisms of clathrin-coated vesicle formation: in addition to the canonical CPs that form *de novo* (Figure 6A) and CPs internalized from the edges of plaques (Figure 6B), for the first time, we report a new mechanism where endocytic vesicles form by dissociation of giant coated pits (GCPs) (Figure 6C). GCPs can be orders of magnitude larger than the canonical pits and have longer lifetimes, however, their mechanism of curvature generation is the same, i.e., independent of a flat-to-curved transition taking place at the late stages of coat formation. While the canonical pits are internalized as a whole, GCPs are internalized by splitting into multiple fragments.

In the osmotic swelling experiments, GCP formation is observed within the first 15 min of the hypo-osmotic treatment. After this point, membrane tension is expected to start converging back to the original levels as the osmotic swelling is temporary and endocytic clathrin dynamics recover from the inhibitory effects of increased membrane tension within ∼30 min after the hypotonic treatment (Dai et al., 1997; Ferguson et al., 2017; Willy et al., 2021b). As the membrane tension reduces, forces provided by actin filaments may increase the curvature of the coat more efficiently if the underlying membrane ceases to support the rigidity of the coat. As demonstrated by our simulations, increased curvature gives rise to increased strain at the edge regions of coat fragments, and consequently, may result in breaks in the clathrin lattice (Figure 5). On the other hand, such a recovery in membrane tension does not take place after cholesterol depletion (Biswas et al., 2019), which may be another reason why GCP splitting events were not observed in MβCD treated cells.

Considering the effects of high membrane tension on the size and curvature of clathrin-coated structures (Saleem et al., 2015; Ferguson et al., 2017; Willy et al., 2021b), we concluded that the formation of GCPs is a result of tension building up on the amnioserosa and lateral epidermis tissues during the dorsal closure of the *Drosophila* embryo (Saias et al., 2015; Hayes and Solon, 2017). The dorsal closure of *Drosophila* embryos is driven by the mechanical forces propagating from the amnioserosa tissue (Kiehart et al., 2017). We believe the formation of GCPs at the neighboring epidermis tissue is induced by the increasing apical surface tension with the progression of dorsal closure (Saias et al., 2015; Hayes and Solon, 2017). The reason GCPs were not observed in the aminoserosa tissue may be related to the pulsed constrictions and relaxations taking place at the apical surface of individual amnioserosa cells during dorsal closure (Solon et al., 2009). The period of these oscillations, which are expected to modulate the tension on the apical surface, may be too short for formation of the GCPs.

*Drosophila* hemocytes execute important non-immune functions, such as clearance of apoptotic debris, during embryo development (Wood and Jacinto, 2007). Starting from stage 10 of embryogenesis, they disperse from the head mesoderm and populate the entire embryo. They are characterized by thin lamellae that allow them to efficiently migrate through cells and tissues (Supplementary Video S3) (Tucker et al., 2011; Willy et al., 2017). The clathrin plaques we observed in hemocytes may facilitate cell migration by serving as adhesion platforms interacting with the vitelline membrane of the embryo (Figure 4) (Lock et al., 2019).

Finally, our *in vivo* data acquired from developing *Drosophila* embryos suggest that GCPs are physiologically relevant. However, formation of GCPs is very sparse (∼4.5 × 10^−3^/µm^2^) in comparison to the canonical CPs (∼0.76/µm^2^), suggesting that they only form under extreme mechanical conditions such as increased local membrane tension. Considering the effects of the cargo size on the geometry and dynamics of endocytic clathrin-coated structures, we expect clustering of ligands, or their receptors may also give rise to formation of endocytic hotspots that contain GCPs (Willy et al., 2021b). Future studies should shed more light on the exact roles played by GCPs at the organismal level.

## 4 Materials and methods

### 4.1 Fluorescence live cell imaging

Genome-edited AP2-eGFP expressing human breast cancer SUM159 cell lines (eGFP incorporated at the C-terminus of the σ2 subunit of AP2) were a kind gift of Dr. Tomas Kirchhausen (Harvard Medical School) (Aguet et al., 2016). Cells were grown at 37°C and 5% CO_2_. SUM159 cells complete media consisted of F-12/Glutamax (Thermo Fisher Scientific), supplemented with 5% fetal bovine serum (Gibco), 100 U/mL penicillin and streptomycin (Thermo Fisher Scientific), 1 μg/ml hydrocortisone (H-4001; Sigma-Aldrich), 5 μg/ml insulin (Cell Applications), and 10 mM 4-(2-hydroxyethyl)-1- piperazine-ethane-sulfonic acid (HEPES), pH 7.4. Complete L15 media (Thermo Fisher Scientific) supplemented with 5% serum and 100 U/ml penicillin and streptomycin was used during live-cell imaging, as it supports cell growth in environments without CO_2_ equilibration. In actin inhibition assays, cells were treated with 1 μM Jasplakinolide (Alexis Biochemical) for 10 min and imaged with a spinning disk confocal microscopy setup built on an Eclipse TI-E microscope (Nikon Instruments Inc.) equipped with a perfect focusing system (PFS), a temperature-controlled chamber, CSU-W1 spinning disc unit (Yokogawa Electric Corporation), a 100× objective lens (Nikon CFI Plan-Apochromat Lambda, NA 1.45), an Electron Multiplying Charge Coupled Device (EMCCD) camera (iXon DU897 Ultra; Andor Technology), and 488- and 640-nm excitation lasers with 100 mW of nominal power. Images were acquired at a rate of 0.25–0.5 Hz with a laser exposure of 100 ms per frame. Image acquisition was done using NIS Elements software.

Cell squeezing experiments were performed as described before (Ferguson et al., 2017). Briefly, a micromanipulator (Narishige MMO-202ND, Narishige MMN-1) controlled polydimethylsiloxane (PDMS) brick was used to press down on cells until the maximum level of squeezing is signaled by inhibition of clathrin coat activity.

### 4.2 Structured illumination microscopy (SIM)

The schematic of the home-built high NA SIM system is shown in Supplementary Figure S1. The beams from 488 nm to 561 nm (300 mW, Coherent, SAPPHIRE LP) lasers are collinearly combined. An acousto-optic tunable filter (AOTF; AA Quanta Tech, AOTFnC-400.650-TN) is used to switch between them and to adjust the illumination power. After passing through the AOTF, the beam is expanded and sent to a phase-only modulator (Kner et al., 2009), which is used to diffract the beam. The phase-only modulator consists of a polarizing beam splitter, an achromatic half-wave plate (HWP; Bolder Vision Optik, BVO AHWP3), and a ferroelectric spatial light modulator (SLM; Forth Dimension Displays, QXGA-3DM-STR).

For high NA SIM acquisitions, nine grating patterns consisting of 3-orientations and 3-phases are displayed on the SLM. The diffracted beams then pass through an azimuthally patterned achromatic half-wave plate (Azimuthal HWP; Bolder Vision Optik), which consisted of three pairs of segments with custom designed fast axis orientations and the linear polarization of the diffracted light from the grating patterns is rotated to the desired s-polarization (Guo et al., 2018). The beams then pass through a mask in order to filter out undesired diffractions and pass only ±1 diffraction orders, which are then relayed onto the back focal plane of the high numerical aperture (NA) objective (Olympus APO ×100 1.65 OIL HR 0.15; a generous gift from Paul Selvin, University of Illinois at Urbana-Champaign) mounted on an inverted Eclipse TI-E microscope (Nikon instruments Inc.). The incidence angle onto the interface of the specimen and coverslip (V-A Optical Labs, SF-11) is adjusted by changing the periodicity of the SLM pattern.

For each frame two SIM acquisitions are taken at two incidence angles which are slightly lower (grazing incidence illumination) or higher (TIR illumination) than the critical angle. The fluorescent emission generated by the applied excitation pattern of each phase and orientation is collected by the same objective and focused by a tube lens onto an EMCCD camera (Andor, iXon Ultra 897). The acquired nine raw images are reconstructed into a super-resolution image based using a previous algorithm (Wiener coefficient = 0.001) (Huang et al., 2018). The cells were left in the incubator for 3 h to allow complete spreading before starting imaging using 488 nm illumination at 2 frames/sec with 20 msec acquisition time.

TIRF-SIM imaging assays of live *Drosophila* embryos and SUM159 cells treated with hypotonic shock and cholesterol depletion were conducted at the Advanced Imaging Center (AIC) of the Janelia Research Campus using a lower NA objective (Olympus UAPON ×100 1.49 OTIRF) as described before (Willy et al., 2021b). This system was built using an inverted microscope (Axio Observer, ZEISS) equipped with a sCMOS camera (Hamamatsu, Orca Flash 4.0). Images were acquired at rates ranging from 2 s/frame to 6 s/frame using 20 ms exposure time.

UAS/GAL4 system was utilized to generate *Drosophila* embryos expressing clathrin-mEmerald as described before (Willy et al., 2021b). After collection, the embryos were aged for 10–12 h at 25°C (corresponding to stage 14 of embryogenesis). The TIRF-SIM imaging was conducted at 22°C after the dechorionated embryos were mounted on coverslips and immersed in halocarbon oil.

### 4.3 Structural analysis and 3D tracking of clathrin-coated structures

The radial averages shown in Figure 3 are calculated using a methodology described elsewhere (Willy et al., 2021b). Briefly, TIRF-SIM images of clathrin pits were upscaled by a factor of 10 using bicubic interpolation. The radial average is then obtained by averaging the intensity profiles along cross sections that are concentric with the center of the structure and separated by 30^o^ increments.

For each frame in the 3D time-lapse movie, max intensity value is picked out of all z stacks at each x-y position to form a 2D movie that contains the maximum intensity. The clathrin traces are then tracked using the TraCKer algorithm (Ferguson et al., 2016) on the 2D maximum intensity movie. TraCKer uses an algorithm based on Mexican hat function to detect traces. For each time point in detected traces, axial (z) position is determined by calculating the center of mass across all z stacks at tracked x-y position. A trace-wide axial position is assigned by calculating the mean z position of all time points in the trace.

Growth rates (slopes) are extracted from the intensity values on the tracked traces as described before (Ferguson et al., 2016). Trace intensities are normalized by subtracting a constant background value. Traces with lower than 12s are discarded. For each 12 s interval, a least-square fit is applied to determine the slope at each time point of the trace.

### 4.4 Simulations of clathrin-coated structures

We start with a clathrin complex consisting of a pentagon surrounded by a honeycomb lattice, as shown in Supplementary Figure S2. The lattice as shown lies in a plane. We then introduce a set of terms contributing to overall energy that, when minimized, leads to the deformation of the lattice into a dome-like structure. The contributions to the energy of the complex are.

1. The energy associated with the angle between adjacent edges of a hexagon
2. The energy associated with the length of a bond
3. The “pucker angle” energy

We address each energy separately below.

#### 4.4.1 The energy associated with the angle between adjacent edges of a hexagon

Consider a given vertex and the three edges that attach to it, as shown in Supplementary Figure S3.

If the edges lie in a plane, and all the angles are equal, then $\theta_{1}=\theta_{2}=\theta_{3}=\frac{2\pi }{3}$ . However, if all angles are the same and are less than $2\frac{\pi }{3}$ , then the vertices cannot lie in a plane. In this case the resulting configuration looks like one of the vertices and three of the edges of a tetrahedron. The energy that favors a departure from planar geometry is introduced via the scalar product of adjacent edges attached to a vertex. Let ${\hat{e}}_{i}$ ( 1≤i≤3 ) be the three unit vectors parallel to the edges incident on a given vertex with tails on the vertex. We construct the energy so that it favors a configuration in which ${\hat{e}}_{i}\cdot {\hat{e}}_{j}=\cos\left(\theta_{0}\right)$ . We adopt the following form for this energy,

$$E_{1}=k_{b}\underset{i,j}{\sum }({\hat{e}}_{i}\cdot {\hat{e}}_{j}-\cos\left(\theta_{0}\right){)}^{2}$$ (A.1)

where the sum is over the nearest neighbor edges, $\theta_{0}$ is the preferred angle between two edges and $k_{b}$ is the bending rigidity. The larger $k_{b}$ is the more important the bending energy.

#### 4.4.2 The energy associated with the lengths of the edges

In the case of the honeycomb lattice in a plane, all edges have the same length. When the lattice acquires a curvature, it becomes necessary to add an energy consistent with the desired lengths of the edges. We will assume an equilibrium (preferred) length of $l_{0}$ . We introduce the stretching energy favoring that length through the expression

$$E_{2}=k_{s}\underset{i}{\sum }(l_{i}^{2}-l_{0}^{2}{)}^{2}$$ (A.2)

where the sum is over link index i , and $l_{i}$ is the length of the link number i . Again, the larger the stretching modulus $k_{s}$ , the more important this contribution to the total energy to be minimized.

#### 4.4.3 The “pucker angle” energy

This energy is based on the volume associated with a local curvature. Consider the triplet of edges shown in Supplementary Figure S3. We can think of this as a projection of three sides of a parallelepiped, shown in Supplementary Figure S4.

If the three edges incident on the vertex shown as a dot in the figure are ${\vec{v}}_{1}$ , ${\vec{v}}_{2}$ and ${\vec{v}}_{3},$ then the volume of the parallelepiped is equal to $\left|{\vec{v}}_{1}\cdot \left({\vec{v}}_{2}\times {\vec{v}}_{3}\right)\right|$ . An important feature of the triple product is that with the absolute value lines removed, it can be positive or negative. Specifying a value of the triple product gives us a set of four vertices—a central vertex surrounded by three nearest neighbors—that produce a positive or negative curvature. If the three edges in this complex are coplanar, then the triple product, and hence the curvature, is zero. It is possible then to introduce an energy based on the triple product favoring a specific local curvature. We note that that there are other ways to include the curvature energy in the model (Panahandeh et al., 2022) but we found that this is the most appropriate for a hexagonal or pentagonal lattice.

Calling this triple-product-based curvature $C\left({\vec{v}}_{1},{\vec{v}}_{2},{\vec{v}}_{3}\right)$ , or $C_{i}$ for short, the energy is of the form

$$E_{3}=k_{p}\underset{i}{\sum }{\left(C_{i}-C_{0}\right)}^{2}$$ (A.3)

where $C_{0}$ is the preferred value of the triple product or curvature. This is directly related to the pucker angle in (den Otter and Briels, 2011). The positive coefficient $k_{p}$ controls the importance of this contribution.

#### 4.4.4 The minimization procedure

We use a straightforward steepest descent method. Given a parameter-dependent energy of the form $E\left(p_{1},p_{2},\ldots \right)$ , where $p_{i}$ is the position of the vertex *i*, we introduce a time-dependence, so that $p_{i}\to p_{i}\left(t\right).$ Then, we solve the equations of motion

$$\frac{{dp}_{i}\left(t\right)}{dt}=-\gamma \frac{\partial E\left(p_{1}\left(t\right),p_{2}\left(t\right),\ldots \right)}{\partial p_{i}\left(t\right)}$$ (A.5)

where the coefficient γ is the relaxation rate. We set it equal to 1 in our calculations. The energy E on the right-hand side of (Eq. A.5) is the sum of the three energies ${E=E_{1}+E_{2}+E}_{3}$ given in (Eq. A.1)-(Eq. A.3), respectively. All adjustable coefficients in those equations are set equal to constant values, with the exception of $C_{0}$ , in (Eq. A.3), the preferred value of the triple product. The parameter $C_{0}$ , slowly increases over time from zero to the final desired value at the end of the simulation. It is assumed and has been numerically verified, that the system responds adiabatically.

In the next section, we show that as the lattice curves more and more, the hexagons at the edge become very deformed and the energy of the system goes so high that the bonds start to break.

#### 4.4.5 Bond breaking

The overall goal of this section is to develop a technique such that when hexagons—and the single pentagon—become sufficiently distorted, then bonds will be broken. The mechanism for determining when this happens depends on the angles between adjacent triskelion bonds. See Supplementary Figure S5.

Note that there are up to four relevant angles between adjacent vertices to consider in determining whether or not the central bond breaks or remains intact. The basis for the determination of whether or not a bond is broken is the expression on the right-hand side of (Eq. A.1), with the coefficient $k_{b}$ set equal to one and the preferred angle $\theta_{0}$ set equal to 2π/3 , so $\cos\left(\theta_{0}\right)=-0.5$ . For each central bond, shown in red in Supplementary Figure S5 we calculate the sum of the two, three or four expressions, depending on the number of external bonds, shown in black. If that sum exceeds a chosen “breakpoint” value, the bond is deemed to have broken. Otherwise, the bond remains intact. The equation for the stress on a given bond is then

$$S_{B}=\overset{i_{m}}{\underset{i=0}{\sum }}{\left(\cos\left(\theta_{i}\right)+0.5\right)}^{2}$$ (A.6)

Where $i_{m}$ is 2, 3 or 4, depending on the type of bond (see Supplementary Figure S5). The criterion for a bond to be broken is $S_{B}\geq b$ , b being the breakpoint, here chosen to be 0.3.

#### 4.4.6 Development of a domed clathrin structure and determining which bonds break

As already noted, we start with a flat sheet consisting of a central pentagon surrounded by hexagons, shown in Supplementary Figure S2. We then set all $p_{i}$ in (Eq. A.5) equal to the coordinates of the vertices in Supplementary Figure S2 as the initial condition for our lattice. This corresponds to the condition when the cell membrane is stretched and is under tension. Then, we calculate the total energy *E* of the lattice using (Eq. A.1) (Eq. A.3) corresponding to the stretching, bending and curvature energies of the lattice.

We recall that right at the beginning of the experiments, the membrane is under tension and keeps the coat curvature low. As the tension goes down, the membrane becomes less stretched (floppier) and it appears that the triskelion network starts to curve.

The value of $C_{0}$ in (Eq. A.3) varies as a function of time, as it quantifies the influence of the membrane substrate in the clathrin complex. The assumption is that the nature of the substrate controls the value of the coefficient $C_{0}$ . A rigid substrate favors a smaller positive value for $C_{0}$ , while as the substrate becomes flaccid $C_{0}$ increases.

There are a number of reasons why $C_{0}$ changes with time. For example, as the tension in the membrane decreases, the membrane below the triskelion lattice becomes floppier and thus occupies more volume. The variation of $C_{0}$ with time could also be due to forces provided by actin polymerization. Regardless of the sources, in our model, we consider the influence of these forces through the change in the value of $C_{0}$ , allowing the flat lattice given in Supplementary Figure S2 to curve.

To mimic the experimental situation at the beginning of the simulations, we set the value of $C_{0}$ equal to zero. This corresponds to the situation where the triskelion network is flat due to the membrane tension. As the membrane tension decreases, the sum of forces on the triskelions is such that they can start to curve. This translates in our model to a non-zero time-dependent $C_{0}$ .

We then solve the equation of motion (Eq. A.1), with parameters in the total energy adjusted so that there is an energetic preference for a constant curvature. We assume that the parameter $C_{0}$ increases linearly with time from zero at t=0 to 0.04 at the final time, which we set equal to $10^{8}$ . All other parameters in the equation for the total energy are given time-independent values. In particular, we set $k_{s}=500$ and $l_{0}=0.64$ in (Eq. A.2), $k_{b}=10^{-8}$ in (Eq. A.2), $k_{p}=100$ in (Eq. A.3) and $\theta_{0}=2\pi /3$ . The last assignment gives rise to a contribution to the energy that favors regular hexagons. The result of our simulations is shown in Supplementary Figure S6. The figure shows the evolution of a clathrin complex grown from a sheet of hexagons surrounding a central pentagon to a dome. The bond stress map $S_{B}$ (Eq. A.6) reveals that the stress is in particular high at the edge. The bonds break if bond stress exceeds the threshold of 0.3. The broken bonds are shown as red lines in Supplementary Figure S6. Note that bond breaking takes place once the dome has fully developed, comparing the values of $S_{B}$ in (Eq. A.6) with the breakpoint of 0.3.

Our simulations are not very sensitive to the values of $k_{s}$ , $l_{0}$ , $k_{p}$ and $k_{b}$ as long as the network is stiff $k_{s}∼k_{p}∼100$ . We varied $k_{b}$ by two orders of magnitude and explored its impact on the final dome structure. The results of our simulations for $k_{b}=10^{-10}$ and $k_{b}=10^{-7}$ are presented in Supplementary Figures S7,8, respectively. As $k_{b}$ increases to $10^{-7}$ , it will be harder to shear the lattice. The stress at the edge decreases but it is still high. This corresponds to very stiff subunits that do not allow the lattice to shear.

We note that den Otter and Briels have also investigated the assembly and disassembly of the clathrin complex (den Otter and Briels, 2011). There are two major differences between the model presented here and theirs. First, they simulate the behavior of individual units consisting of a central vertex and three bent legs that assemble (and disassemble) in the process of forming a clathrin complex. Second, we allow the clathrin lattice’s angles and edges to change, whereas their units are mechanically rigid. Since clathrin shells can assume different sizes depending on the cargo (Wagner and Zandi, 2015; Zandi et al., 2020), we believe that the elastic energies in our model are suited to describe the experiments presented in this paper.

## Data Availability

The raw data supporting the conclusions of this article will be made available by the authors, without undue reservation.
