# Supplementary figures and images for: Endocytosis at extremes: Formation and internalization of giant clathrin-coated pits under elevated membrane tension
Source: Front Mol Biosci. 2022 Sep 21;9:959737. doi: 10.3389/fmolb.2022.959737 (PMC9532848; doi:10.3389/fmolb.2022.959737)

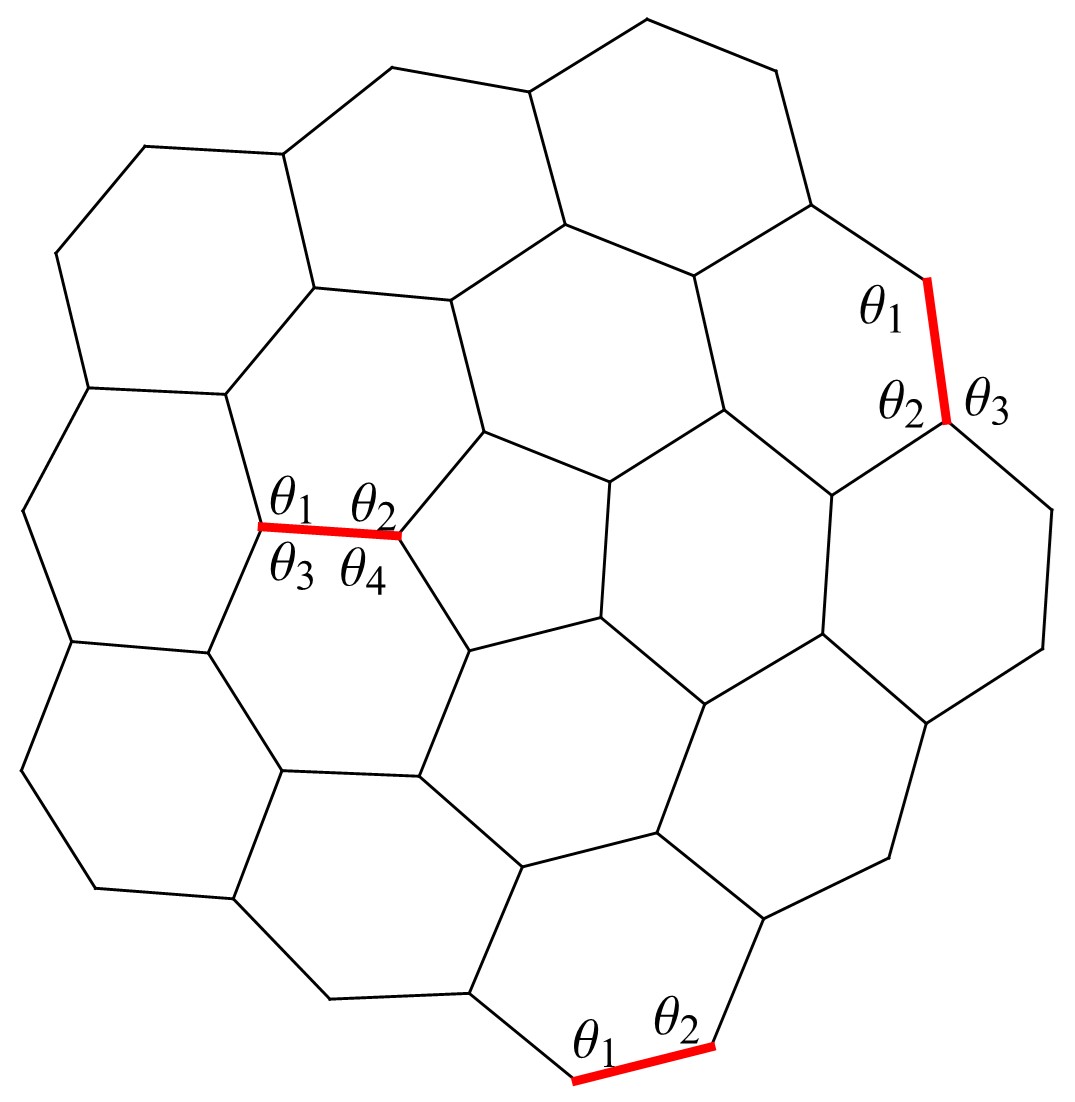

Supplement: Supplementary file 2 [file Image5.jpg]

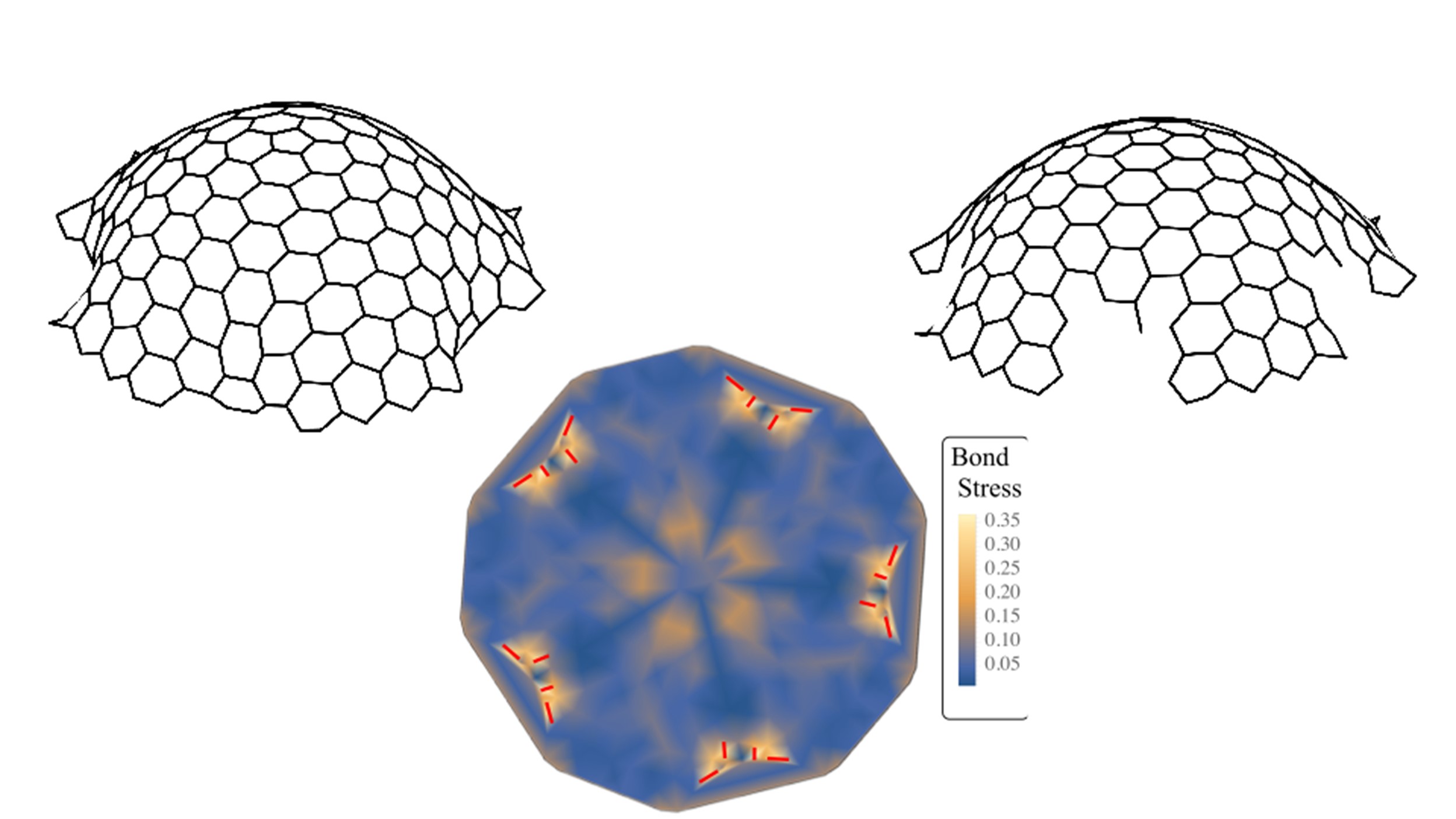

Supplement: Supplementary file 4 [file Image6.jpg]

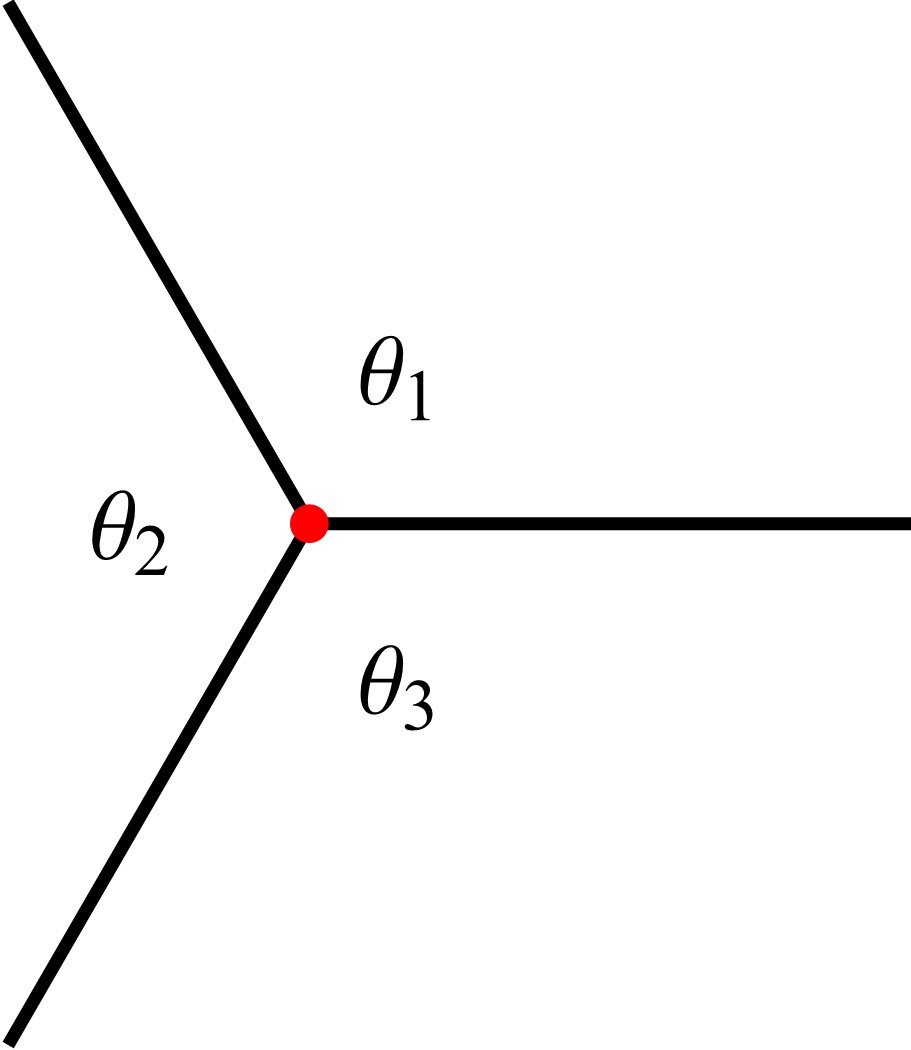

Supplement: Supplementary file 5 [file Image3.jpg]

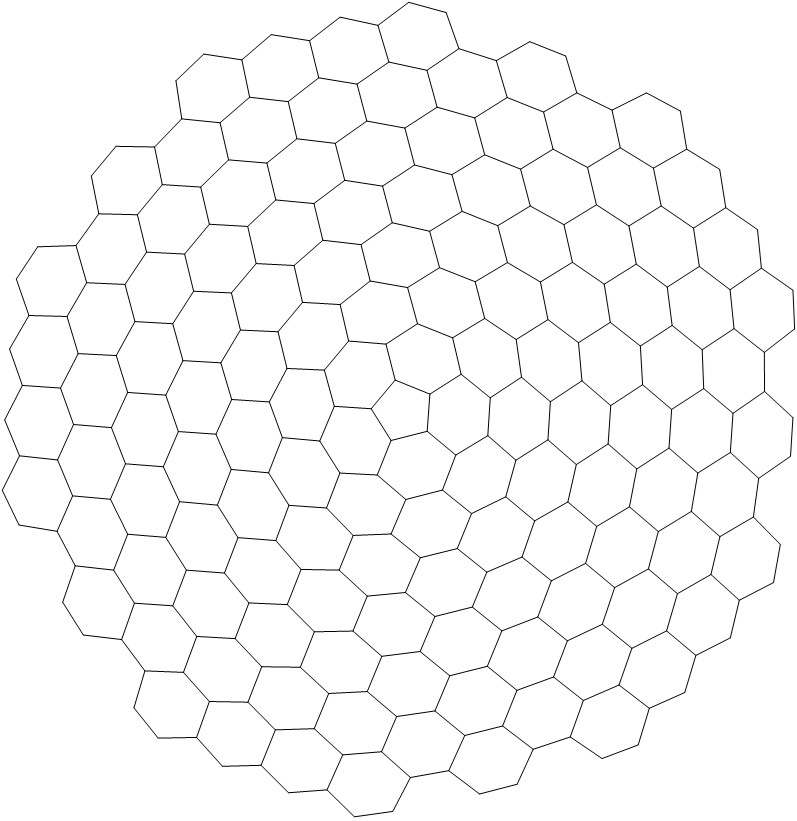

Supplement: Supplementary file 6 [file Image2.jpg]

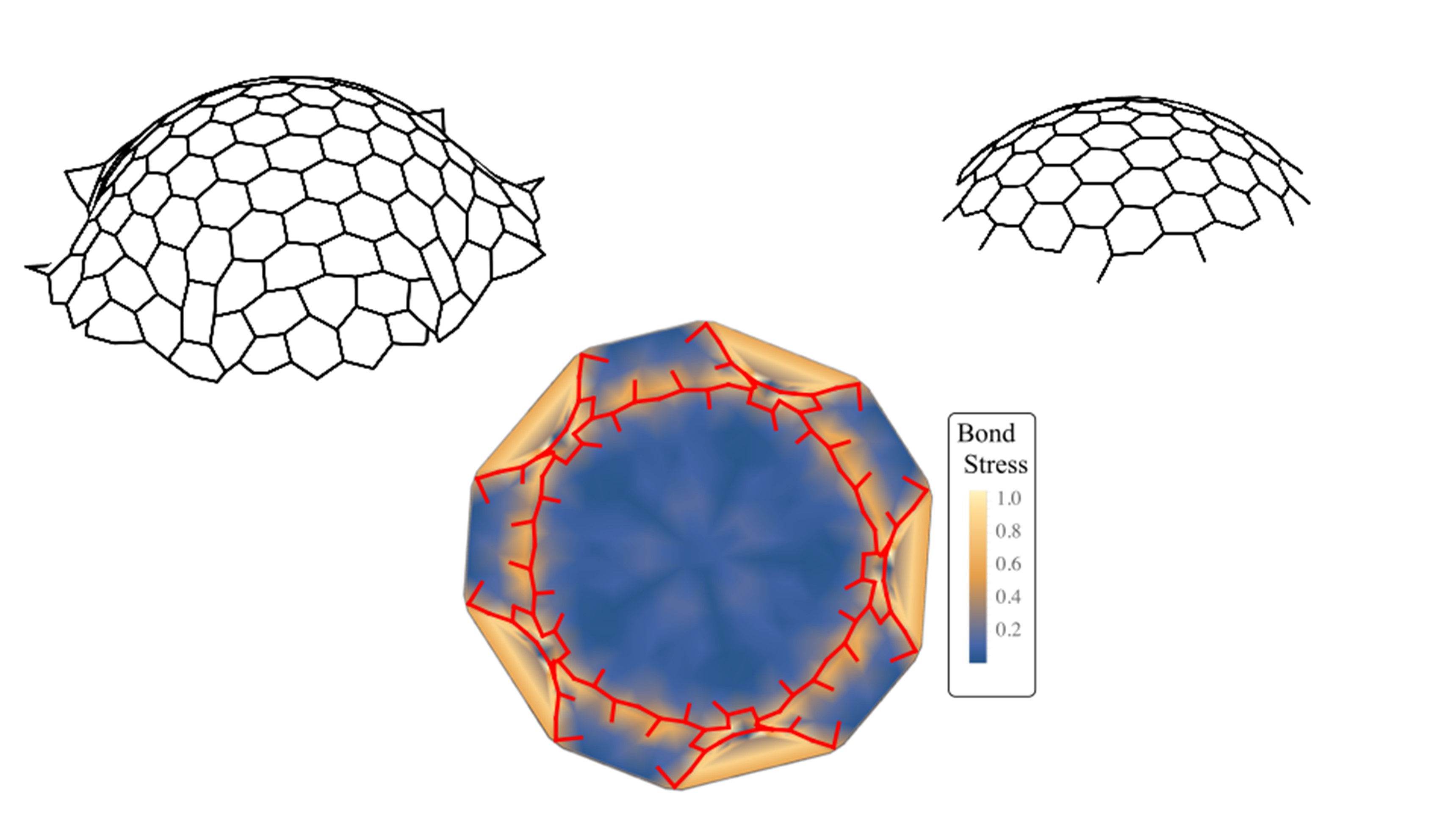

Supplement: Supplementary file 8 [file Image7.jpg]

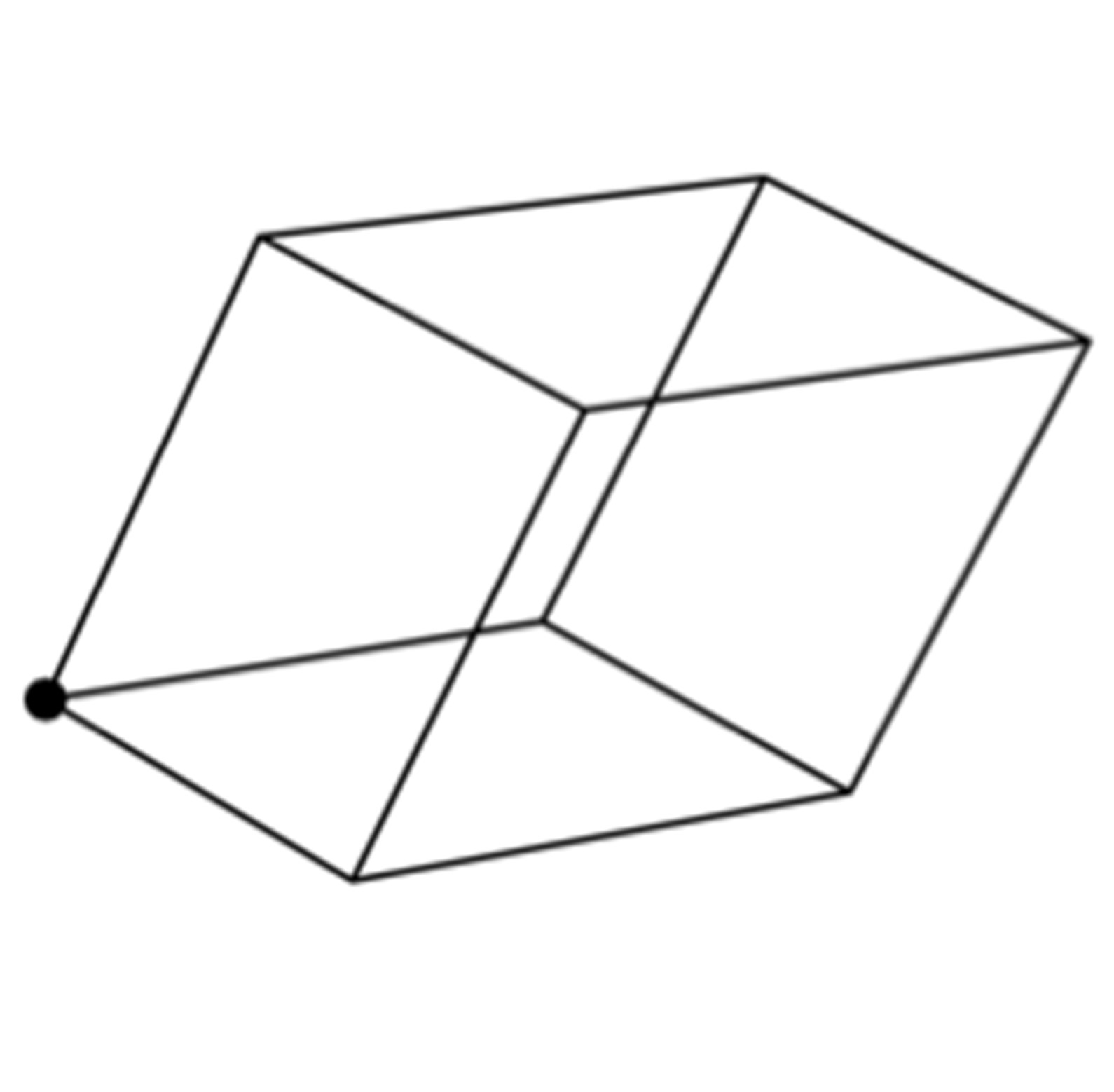

Supplement: Supplementary file 9 [file Image4.jpg]

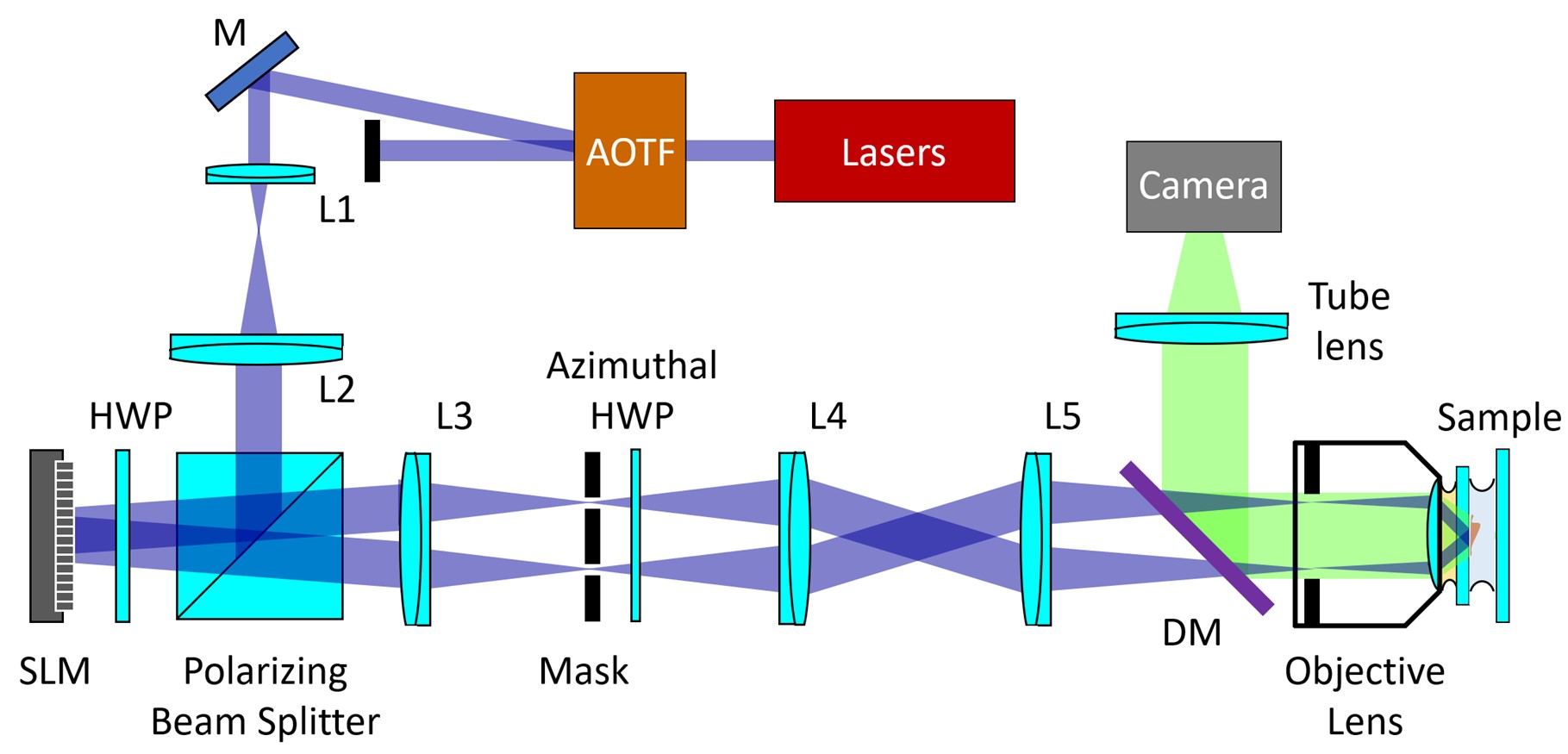

Supplement: Supplementary file 11 [file Image1.jpg]

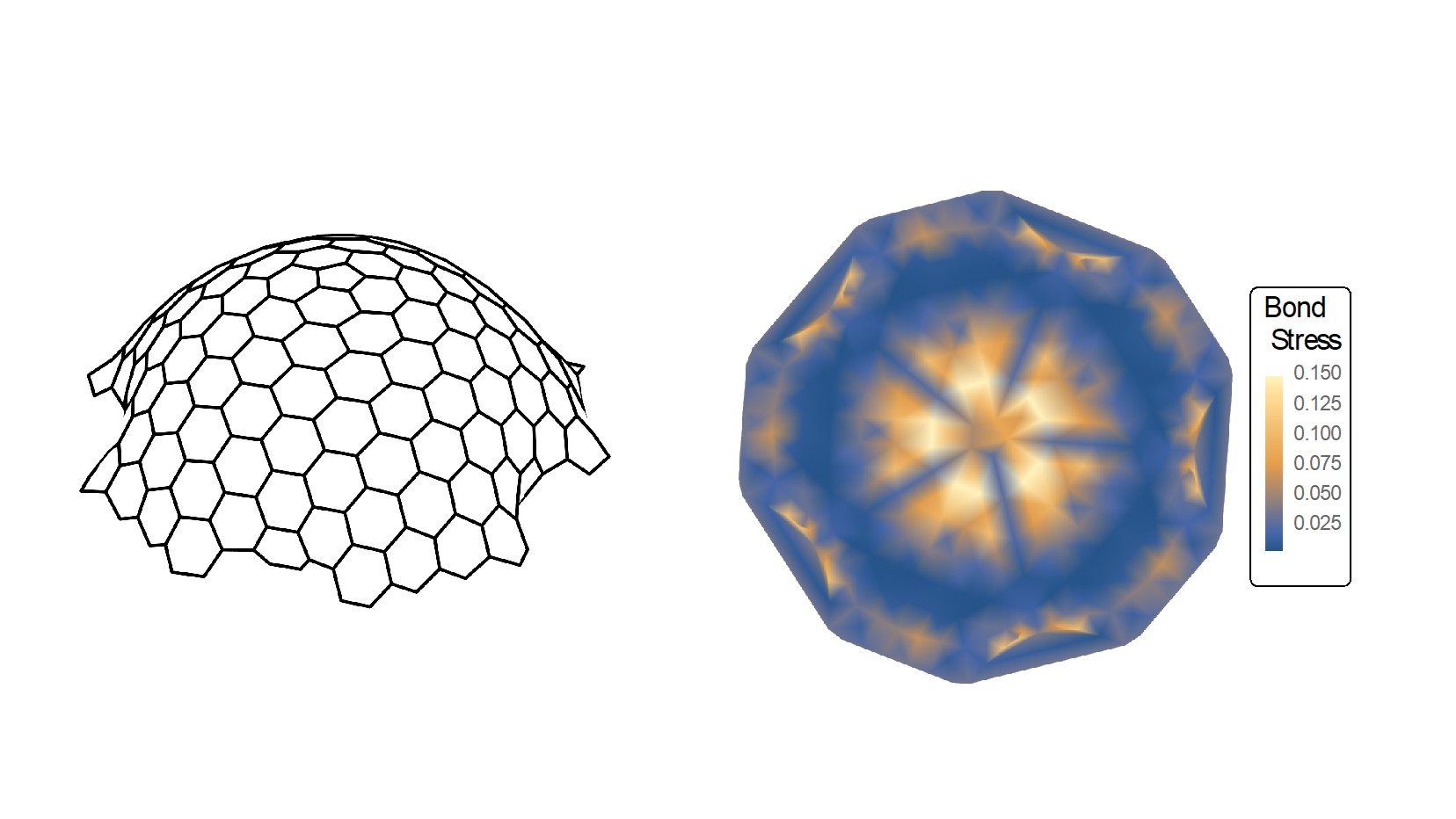

Supplement: Supplementary file 12 [file Image8.jpg]
