# Supplementary material for: Endocytosis at extremes: Formation and internalization of giant clathrin-coated pits under elevated membrane tension
Source: Front Mol Biosci. 2022 Sep 21;9:959737. doi: 10.3389/fmolb.2022.959737 (PMC9532848; doi:10.3389/fmolb.2022.959737)
Supplement: Supplementary file 7 [file Table1.pdf]

Captions of the supplementary material

Supplementary Figure S1. Optical configuration of the home-built structured illumination microscopy (SIM). AOTF: acousto-optic tunable filter; HWP: half wave plate; SLM: spatial light modulator; L1-L5: lenses; M: mirror; DM: dichroic mirror.

Supplementary Figure S2. The initial complex, consisting of a pentagon surrounded by a honeycomb-like lattice. The complex lies in a plane, which means that the hexagons are distorted versions of the regular hexagons in a true honeycomb lattice.

Supplementary Figure S3. A vertex and the three edges attached to it, along with the angles between the edges.

Supplementary Figure S4. The parallelepiped constructed from the three edges incident on the vertex shown as a dot.

Supplementary Figure S5. Examples of a bond, in red which joins two nearest neighbor vertices, shown for a smaller version of the clathrin complex. The two vertices have either one or two additional bonds attached. The relevant angles between adjacent bonds are $\theta_1, \ldots \theta_4$, as shown.

Supplementary Figure S6. Results of simulating the evolution of a clathrin complex grown from a flat sheet of hexagons surrounding a central pentagon according to the equation (A.5) with $\gamma = 1$ and the total energy E=$E_1 + E_2 + E_3$ is calculated using Eqs. (A.1)-(A.3). The parameters used in simulations are $k_s = 500$, $l_0 = 0.64$, $k_b = 10^{-8}$, $k_p = 100$ and $\theta_0 = 2\pi/3$. On the left: the shape of the complex at the end of the simulation. On the right: In the center: the bond stress map S_B, as given by Eq. (A.6), as viewed from above the dome, along with the bonds that break because of bond stress that exceeds the threshold of 0.3. The broken bonds are shown as red lines. On the right: the clathrin complex with the broken bonds removed, along with small detached fragments.

Supplementary Figure S7. Results of the simulation described in the captions of Supplementary Figure 6, except that in this case $k_b = 10^{-10}$. On the left: the complex that evolves. In the center: the bond stress map, along with the bonds that break because of bond stress that exceeds the threshold of 0.3. The broken bonds are shown as red lines. On the right: the clathrin complex with the broken bonds removed, along with small detached fragments.

Supplementary Figure S8. Results of the simulation described in the captions of Supplementary Figure 7, except that in this case $k_b = 10^{-7}$. On the left: the shape of the complex at the end of the simulation. On the right: a plot of the bond stress, S_B, as given by Eq. (A.6), as viewed from above the dome. No bonds are broken as the result of excessive bond stress.

Supplementary Video S1. Examples of GCP formation and dissolution detected upon hypotonic swelling in SUM159 cells genome edited to express AP2-EGFP. Scale bar, 1μm.

Supplementary Video S2. TIRF-SIM movie of clathrin coat dynamics acquired at the amnioserosa tissue of a late *Drosophila* embryo expressing clathrin-mEmerald. Further zoom-in (blue box) shows growth and internalization of an individual clathrin-coated pit (arrowhead).

Supplementary Video S3. TIRF-SIM movie of clathrin coat dynamics acquired at the lateral epidermis of a late *Drosophila* embryo expressing clathrin-mEmerald. The panels below are the zoom-ins corresponding to four GCPs. The 4th GCP gets out of focus due to a hemocyte entering the field of view and migrating between the epidermis and the vitelline membrane.

Supplementary Video S4. Examples of GCPs splitting into multiple fragments at the lateral epidermis of late *Drosophila* embryos. Scale bar, 2μm.
